# Hotspots and Trends in Research on Mental Health of the Rural Elderly: A Bibliometric Analysis Using CiteSpace

**DOI:** 10.3390/healthcare13030209

**Published:** 2025-01-21

**Authors:** Yinzhu Hao, Lei Yang

**Affiliations:** School of Public Health, Hangzhou Normal University, Hangzhou 311121, China; 2020011012013@stu.hznu.edu.cn

**Keywords:** mental health, rural elderly, bibliometric analysis, CiteSpace

## Abstract

Background: This study addresses the need for a systematic analysis of research trends and hotspots in the mental health of rural elderly populations, a field that has gained increasing attention due to the growing global aging population. The previous literature has explored specific issues such as loneliness, depression, and social support, and yet a comprehensive review of research development, key themes, and gaps remains lacking. Methods: Using CiteSpace software (CiteSpace 6.3.R1), this study conducts a bibliometric analysis of 1545 articles from the Web of Science Core Collection and 1793 articles from the China National Knowledge Infrastructure. The analysis identifies key themes, collaboration networks, and the evolution of research focus over time. Results: The results reveal that while Chinese research in this field is highly active and diverse, international collaboration is limited, underscoring the need for stronger global partnerships. Conclusions: Emerging research areas, such as the impact of socioeconomic factors, intergenerational support, and rural healthcare policies, are identified. These findings provide valuable insights into the current state of research and highlight opportunities for future studies and policy-making aimed at improving the mental health and quality of life of rural elderly populations worldwide.

## 1. Introduction

As China’s population continues to age, the number of elderly individuals in rural areas is steadily increasing, and their mental health issues are gaining widespread social attention. The mental health of rural elderly not only impacts their quality of life but is also closely related to the harmony and stability of society as a whole [1]. However, due to factors such as relatively lagging economic development, limited medical resources, and insufficient social support in rural areas, the mental health status of the rural elderly is far from optimistic and requires urgent in-depth research and attention [2,3]. Global aging poses significant challenges to healthcare systems, particularly in rural areas where access to medical services is limited [4]. In China, the mental health problems of rural residents are largely influenced by socioeconomic factors, with lower-income elderly individuals being more prone to depression [5]. Social capital and social integration play crucial roles in the health of elderly migrants [6].

In recent years, research on the mental health of rural elderly populations has shown a notable upward trend globally, reflecting growing academic and social interest in this field. The mental health of rural elderly individuals is influenced by complex factors, such as limited access to medical services, insufficient social support, and challenges arising from urban–rural integration, which exacerbate their psychological vulnerabilities [7,8].These challenges are not confined to China; they are echoed in rural regions worldwide, where disparities in healthcare infrastructure and social systems often leave the elderly at greater risk. Comparative studies across different countries have highlighted the universal relevance of this issue, making it critical to analyze the topic from both domestic and international perspectives.

However, despite the growing interest, a comprehensive and systematic review of the overall development trends, research hotspots, and frontiers in this field remains lacking. Existing studies in developed countries, have explored topics like the role of community-based interventions and digital technologies in rural elderly mental health [9,10]. Meanwhile, developing countries face unique challenges linked to healthcare access and socioeconomic disparities [11,12]. This gap is particularly significant when considering the differences in research focuses, methodologies, and outcomes between Chinese and English-language studies. To address this gap, this paper leverages CiteSpace software, a bibliometric tool with advanced visualization capabilities, to systematically analyze the domestic and international literature.

By revealing research hotspots, knowledge bases, and evolutionary paths across both Chinese and English studies, this research provides an in-depth understanding of the current global research landscape. The study not only identifies critical trends and gaps but also bridges the divide between domestic and international perspectives, offering insights to guide future research directions and inform policy-making on a global scale. This dual perspective ensures that the findings are both locally relevant and internationally significant, contributing to the broader discourse on rural elderly mental health.

## 2. Methods

### 2.1. Research Methodology

In this study, we utilized CiteSpace, a sophisticated bibliometric tool developed by Professor Chaomei Chen, to perform an in-depth analysis of the literature on rural elderly mental health. Compared to other widely used tools like VOSviewer, HistCite, and BibExcel, CiteSpace offers unique capabilities that are particularly beneficial for identifying trends and structures in academic research [13,14,15,16,17,18]. Its advanced features, such as dual-map overlays, citation burst detection, and automated cluster labeling, allow for a comprehensive exploration of research evolution and hotspots.

Moreover, CiteSpace integrates algorithms for cluster analysis and visualization, enabling a nuanced understanding of co-authorship networks, citation linkages, and thematic clusters. These functions surpass the basic visualization and mapping capacities of tools like VOSviewer, which focuses on density and grid maps, or HistCite, which primarily organizes citation histories without deeper thematic analysis.

CiteSpace’s dynamic visualization of temporal trends and its capacity for identifying bursts of citation activity make it particularly suited to uncovering pivotal changes in the research focus over time. This capability ensures a systematic and objective understanding of how the field has evolved and highlights emerging areas of inquiry [19]. By leveraging the most recent version, CiteSpace 6.3.R1, we ensured access to its latest advancements, which further enhance analytical depth and visual clarity.

These advantages underscore CiteSpace’s suitability for this study, as it not only maps the structural framework of the research field but also captures its developmental trajectory and intellectual milestones.

### 2.2. Data Sources

The data for this paper were sourced from the Web of Science (WOS) Core Collection and the China National Knowledge Infrastructure (CNKI) database, with the retrieval period set from 1 January 1991 to 31 December 2023, both of which are highly authoritative and widely recognized in academic research. The Web of Science (WoS), managed by Clarivate Analytics in the USA [20,21], is widely recognized as the world’s most authoritative citation database. Renowned for its stringent indexing standards and extensive multidisciplinary coverage, the WoS Core Collection ensures comprehensive access to high-impact journals across various scientific fields. This reliability makes it a cornerstone for bibliometric analyses, providing a robust foundation for the global scientific literature [22,23,24,25,26,27,28]. Its inclusion in this study guarantees that the analysis encompasses influential, peer-reviewed research with a strong international representation, thereby enhancing the credibility and depth of the findings.

Similarly, CNKI is the leading database for Chinese academic publications, offering extensive access to research across various disciplines. It is particularly valuable for capturing region-specific insights and trends, ensuring that this study includes perspectives and findings unique to Chinese scholars. By combining WOS and CNKI, this research bridges global and regional academic discourses, providing a balanced and thorough examination of the field.

For international research, a search was conducted in the WOS Core Collection using topic keywords, connected by “AND” as follows: Topic: ((TS = ((rural) OR (village))) AND TS = ((elderly) OR (old people) OR (old adults))) AND TS = ((mental health) OR (psychological health)). The search scope was limited to the “Web of Science Core Collection (WoSCC),” with the document type set to “ARTICLE” and the language selected as “ENGLISH”. A total of 4051 valid articles were retrieved and exported in “full record and cited references” format.

For domestic research, the search was conducted in the CNKI database using the following format: Topic: (rural + village + township) AND (elderly + old people) AND mental health. A total of 1894 valid articles were retrieved as the domestic research results. The research records included titles, authors, institutions, keywords, years, and journals. Unqualified documents were excluded, and then the remaining data were analyzed.

Given the linguistic and cultural differences between Chinese and English research contexts, terms were adapted to align with common usage in each dataset. For example, while “rural” and “village” are used interchangeably in WOS, CNKI frequently incorporates “township” (乡镇) to capture the socioeconomic nuances of rural areas. Similarly, terms like “mental health” in WOS are more commonly referred to as ‘psychological health’ or ‘mental well-being’ in the Chinese literature, all of which correspond to the Chinese term “心理健康”. Additionally, “old people” and “old adults” both translate to “老人” in Chinese, representing the elderly population in general.

**Figure 1:**
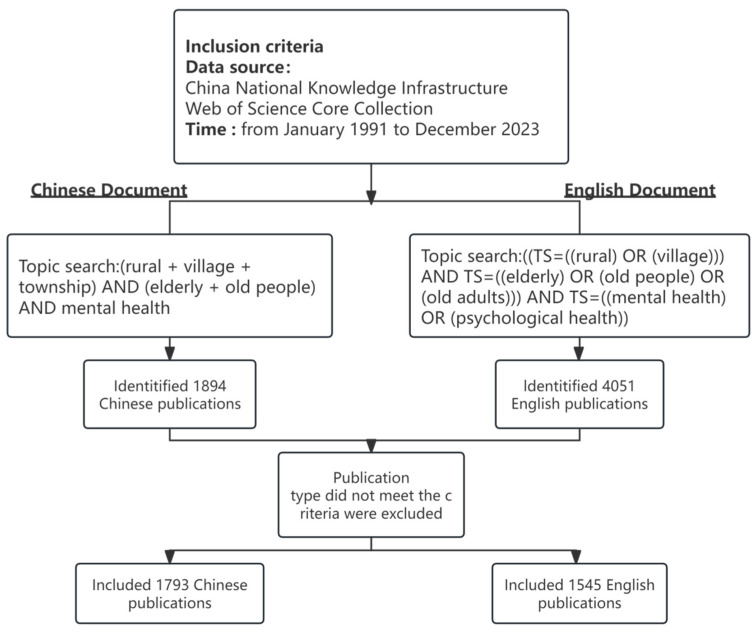
Flow chart of the literature selection.

To ensure the accuracy of the research data, the retrieval process was independently conducted by two researchers, who carried out retrieval, checking, screening, and confirmation. Discrepancies were resolved through consensus discussions, and external validation was performed by a third-party expert during critical phases, ensuring robustness in the methodology. The final optimized dataset for the international literature consisted of 1545 articles, while for the domestic literature, 1793 articles were retained after the final conversion. Details of the filtering process are presented in Figure 1.

## 3. Results

### 3.1. Publication Volume

**Figure 2:**
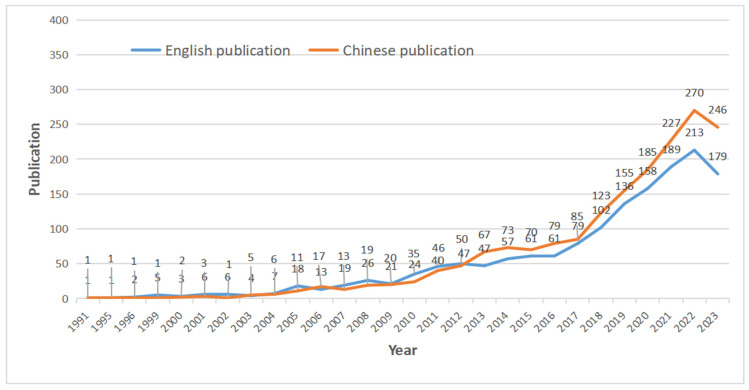
Number of publications related to rural elderly mental health.

Figure 2 shows the trends in English and Chinese publications on rural elderly mental health from 1991 to 2023. The chart clearly demonstrates that Chinese publications have outpaced English publications in recent years. From 1991 to 2013, the number of publications in both languages remained relatively low, with gradual increases over the years. However, from 2014 onward, there was a sharp rise in both English and Chinese publications. Notably, Chinese publications show a steady growth trend after 2017, peaking at 270 articles in 2022, with a slight decrease to 246 articles in 2023. In comparison, English publications saw a dramatic surge after 2016, peaking at 213 in 2022 and slightly decreasing to 179 in 2023. These data indicate that while both English and Chinese research communities have shown increasing interest in rural elderly mental health, Chinese publications have significantly outpaced English publications since 2017, reflecting a stronger national focus on this issue. The higher volume of Chinese publications suggests that the topic is a more prominent research priority in China, particularly in light of the country’s aging population.

### 3.2. Author Collaboration

**Figure 3:**
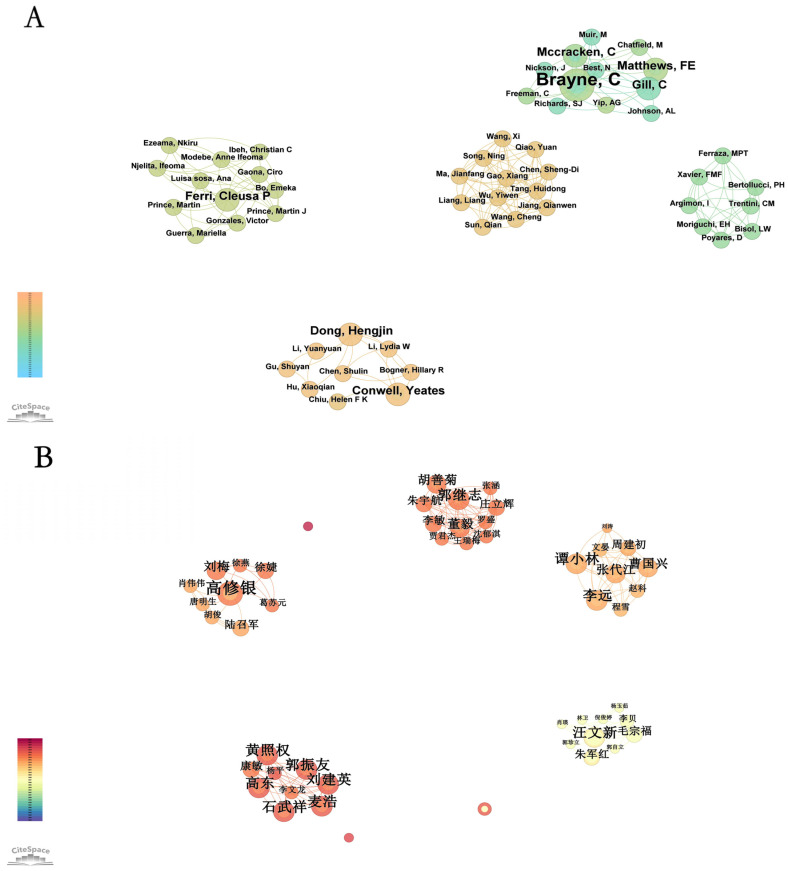
Bibliometric analysis of authors related to rural elderly mental health research: (**A**) English publications, (**B**) Chinese publications.

Figure 3A presents the author collaboration network for the English literature, highlighting key research dynamics in the field of rural elderly mental health. From 2014 to 2023, the English publication volume grew rapidly, driven by influential collaboration networks led by central figures such as Brayne C., Matthews F.E., and Dong Hengjin. These authors, through extensive collaboration, have significantly advanced research in the field. The network also reflects regional and thematic diversity, illustrating distinct research directions across groups. This trend aligns with the steady increase in publication volume, underscoring the field’s growing international prominence and the role of collaborative efforts in driving impactful research.

Figure 3B illustrates the collaboration network among Chinese authors in rural elderly mental health research. The network reveals several core research clusters, including those centered around Gao Xiuyin, Huang Zhaoquan, and Wang Wenxin. These clusters exhibit strong internal collaboration and a sustained academic output, demonstrating their significant influence in advancing specific research directions. However, cross-cluster collaboration remains limited, suggesting a degree of research fragmentation. This independence among clusters reflects diverse focuses and methodologies, while also highlighting opportunities for enhancing inter-cluster cooperation to drive innovation and foster more comprehensive research progress.

In summary, the field of rural elderly mental health is characterized by core author-led collaboration networks that drive research progress and demonstrate thematic diversity. Strengthening cross-cluster and cross-national collaboration could unlock greater innovation and accelerate the field’s development, addressing both regional and global challenges.

### 3.3. Institutional Collaboration

**Figure 4:**
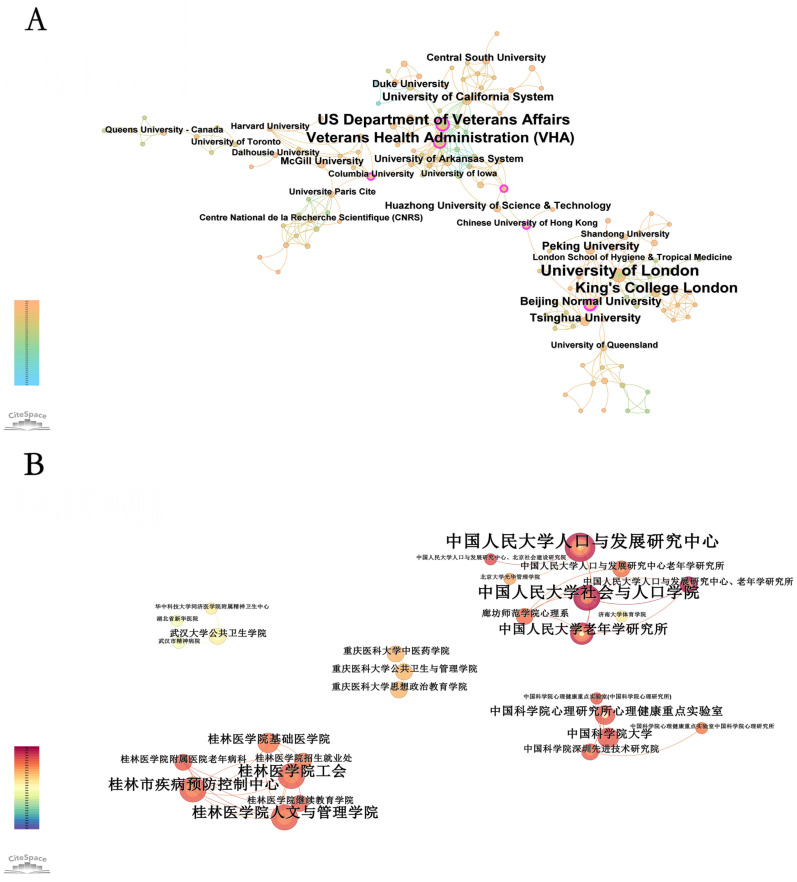
Knowledge map of institutions related to rural elderly mental health research: (**A**) English publications, (**B**) Chinese publications.

Figure 4A displays the co-occurrence network of institutions for the English literature. This figure reveals a global collaborative network structure in the field of mental health of the rural elderly, with several core institutions playing key roles in international cooperation. The U.S. Department of Veterans Affairs (VHA) and the Veterans Health Administration (VHA) are centrally positioned in the network, indicating their dominant role and profound impact on international collaboration in this field. These institutions not only hold a significant position in domestic research within the United States but also connect numerous research institutions from other countries through extensive international cooperation.

At the same time, leading British institutions such as the University of London and King’s College London are also prominent in the collaboration network, particularly forming strong partnerships across Europe and globally. This indicates that the UK is not only an important academic center in this research area but also contributes to research development through close collaboration with other countries.

The network also includes major research institutions from other countries, such as Peking University, Tsinghua University, and the University of Toronto. These institutions form tight collaboration networks within their respective regions and extend their research influence through cross-regional cooperation. Chinese research institutions, in particular, show close cooperation with other East Asian countries, creating relatively independent regional collaboration clusters while also participating in broader international collaboration.

Figure 4B illustrates the co-occurrence network of institutions for the Chinese literature. The analysis of this network reveals notable centralization and diversification in the collaboration structure within the field of mental health of rural elderly in China. Core research institutions such as the Population and Development Research Center at Renmin University of China and the Chinese Academy of Sciences occupy important positions in this field. These institutions not only have significant academic influence but also reinforce their central position in the research network through close collaboration with other institutions. The figure shows that the collaboration networks of these core institutions are dense, forming several high-density node groups that represent frequent collaboration and rich research outputs.

Additionally, research institutions from different regions form relatively independent collaboration clusters, reflecting the diversity of research themes. For example, a cluster centered around Guilin Medical University and its affiliated institutions focuses on medical management and disease prevention, indicating that research on mental health of rural elderly extends beyond sociology and demography to include medicine and public health. This thematic diversity further broadens the depth and breadth of research in this field.

Overall, these research institutions, through regional and interdisciplinary cooperation, have established robust collaboration networks that drive the ongoing development of research on mental health of the rural elderly. Future efforts to strengthen cross-regional and interdisciplinary collaboration are expected to further enhance the research level and global impact of this field.

Table 1 compares the academic influence and collaborative network characteristics of institutions in the field of mental health of the rural elderly, based on both the English and Chinese literature. The table lists the top five institutions for both the English and Chinese literature, analyzing their publication count (Count), centrality (Centrality), year of first publication (Year), and affiliated institutions (Institutions).

In the English literature, the University of London ranks first with 79 publications, first publishing in 1999, and a centrality of 0.06. This indicates the institution’s dominant position in publication volume, but its international collaborative breadth is relatively limited. The U.S. Department of Veterans Affairs and the Veterans Health Administration follow, with 42 and 29 publications, respectively, and centralities of 0.15 and 0.13. This highlights their core positions in the collaboration network, especially in international cooperation. King’s College London ranks third with 41 publications and a centrality of 0.06, indicating a high research influence but a need for further expansion in collaborative networks. Notably, Peking University, with 25 publications and a centrality of 0.22, is ranked fifth, reflecting its growing impact in international collaborations, particularly in cross-national research.

In contrast, institutions in the Chinese literature show high publication volumes but lower centrality, suggesting their research is primarily focused on domestic collaborations. Shandong University leads with 65 publications, but with a centrality of 0, indicating significant domestic influence but an undeveloped international collaboration network. Southwestern University of Finance and Economics and Zhongnan University of Economics and Law rank second and third with 39 and 24 publications, respectively, but also have a centrality of 0, suggesting their research is concentrated domestically, with limited international engagement. Jilin University and Huazhong University of Science and Technology, with 24 and 22 publications, respectively, are also among the top five but show a centrality of 0, reflecting a limited collaborative scope.

In summary, institutions in the English literature generally display stronger international cooperation capabilities and network centrality, indicating their key roles in global research collaboration. On the other hand, institutions in the Chinese literature, while prominent in publication volume, have collaborative networks primarily confined to domestic contexts, with limited international breadth and depth. This reflects differences in resource sharing, collaboration mechanisms, and global impact between institutions in China and abroad, and suggests that Chinese research institutions need to further expand international cooperation to enhance their global academic influence in this field.

### 3.4. National Collaboration

**Figure 5:**
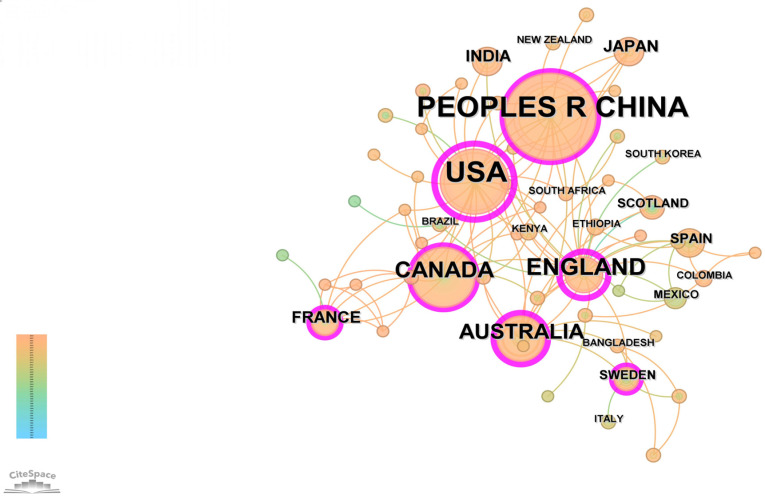
Knowledge map of countries related to rural elderly mental health.

Figure 5 presents the international collaboration network in the field of rural elderly mental health, revealing the academic influence and cooperative relationships among countries. The diagram shows that China and the United States are the core countries in this field, with the largest nodes representing the highest publication volumes and academic influence. These two countries not only lead in research output but also form a dense global research network through extensive international collaborations, maintaining close connections with multiple countries, underscoring their key roles in global cooperation.

In addition to China and the USA, countries such as the United Kingdom, Canada, Australia, and France also play significant roles in this field. Although their nodes are relatively smaller, they have dense collaborative networks with other nations, especially among English-speaking countries, reflecting frequent cross-national cooperation. Regional cooperation is also evident among Asian and African countries, with Japan, India, and South Korea showing close collaboration within Asia, while South Africa and Kenya highlight the diversity of research in the African context through their participation in the global network.

Overall, the co-occurrence diagram reflects a highly internationalized research collaboration network, particularly in Europe, North America, and the Asia-Pacific region, forming extensive and dense cooperative relationships. China has increasingly become a central player in international cooperation in recent years, driving the rapid development of research on rural elderly mental health through collaborations with multiple countries. Future efforts to strengthen cross-regional and cross-national collaborations, particularly among emerging countries, are expected to further enhance the breadth and depth of research in this field and promote global knowledge exchange and sharing.

Table 2 details the academic contributions and international collaboration network influence of the top five countries in the field of rural elderly mental health research based on the English literature. China leads with 342 publications, indicating strong research output in this field. However, despite its leading publication volume, China’s centrality is 0.13, suggesting a relatively limited intermediary role in the international collaboration network, with cooperation primarily confined to specific circles and not yet fully integrated into the global mainstream academic network.

The United States ranks second with 226 publications, but its centrality is 0.64, significantly higher than other countries, highlighting its core position in the global research collaboration network. Since its first relevant publication in 1991, the U.S. has built a strong academic foundation through long-term accumulation and extensive international collaboration, becoming a global research hub in this field.

The United Kingdom ranks third with 172 publications and has the highest centrality of 0.78 among all countries. This indicates that the UK plays a crucial intermediary role in international collaboration, gradually becoming a key node in the global research network through close international cooperation since its first publication in 1997.

Canada and Australia rank fourth and fifth with 96 and 82 publications, respectively. Canada’s centrality is 0.19, with its first publication in 1999, reflecting its tight collaboration network in North America and other English-speaking countries. Australia’s centrality is 0.25, with its first publication in 2003, indicating its significant position in global collaboration, though its influence is relatively smaller compared to the U.S. and the UK.

In summary, these countries differ in their academic accumulation and the breadth and depth of their international collaboration networks in the field of rural elderly mental health research. While China leads in publication volume, its influence in the global collaboration network still has room for growth. In contrast, the U.S. and the UK, with higher centrality, are deeply integrated into the global academic collaboration network and lead international research efforts. Canada and Australia also play important roles through regional collaborations, though their global impact is relatively smaller. Strengthening China’s intermediary role in international collaboration and further integrating into the global academic network will enhance its international influence in this field.

### 3.5. Co-Cited Journals

Table 3 lists the top ten most-cited English journal articles in the field of rural elderly mental health research, highlighting their academic impact and importance across different research topics.

The article ranked first is “UCLA Loneliness Scale (Version 3): Reliability, validity, and factor structure”, published in the *Journal of Personality Assessment* in 1996, which has been cited 3119 times. This foundational paper discusses the reliability, validity, and factor structure of the UCLA Loneliness Scale, a widely used tool for assessing perceived loneliness across various populations [29]. The scale’s robustness in measuring loneliness makes it a critical resource in elderly mental health research.

Second is the 2005 article “Twelve-month use of Mental Health Services in the United States—Results from the National Comorbidity Survey Replication,” published in *Archives of General Psychiatry*, with 1987 citations. This paper provides an in-depth analysis of the use of mental health services in the U.S., finding that approximately 41% of individuals with diagnosable mental disorders utilized mental health services within a year [30,31].

The third article, published in *American Journal of Geriatric Psychiatry* in 2006, titled “Definitions and predictors of successful aging: A comprehensive review of larger quantitative studies”, has been cited 829 times. It offers a comprehensive review of definitions and predictors of successful aging, focusing on aspects such as disease absence, maintained physical and cognitive functions, and active life participation [32].

The fourth-ranked article, “Prevalence, treatment, and associated disability of mental disorders in four provinces in China during 2001-05: An epidemiological survey,” published in *Lancet*, has been cited 815 times. This paper provides epidemiological survey results from four provinces in China, revealing a high prevalence of mental disorders with significant functional impairment but low treatment rates [33].

Articles ranked fifth through tenth, published in top journals such as *Lancet Public Health*, *Sleep*, the *Journal of the American Geriatrics Society*, *World Psychiatry*, and *Physical Therapy*, cover a broad range of topics including elderly sleep disorders, epidemiological studies, and functional capacity in living spaces. These studies not only broaden the research scope in rural elderly mental health but also span multiple disciplines such as geriatric medicine, psychiatry, public health, and rehabilitation medicine, reflecting the field’s diversity and complexity.

In summary, these highly cited articles represent the forefront and core issues in rural elderly mental health research. They play a significant role in mental health studies and provide a solid theoretical and empirical foundation for interdisciplinary research. The widespread citation of these works highlights their critical role in advancing global research and guiding future studies.

**Table 3:** Top 10 co-cited references in rural elderly mental health research.

Rank	Frequency	Year	Article Title	Journal Title
1	3119	1996	UCLA Loneliness Scale (Version 3): Reliability, validity, and factor structure [29]	*Journal of Personality Assessment*
2	1987	2005	Twelve-month use of Mental Health Services in the United States: Results from the National Comorbidity Survey Replication [30]	*Archives of General Psychiatry*
3	829	2006	Definitions and predictors of successful aging: A comprehensive review of larger quantitative studies [32]	*American Journal of Geriatric Psychiatry*
4	815	2009	Prevalence, treatment, and associated disability of mental disorders in four provinces in China during 2001-05: An epidemiological survey [33]	*Lancet*
5	703	2009	Health in South Africa: The burden of non-communicable diseases in South Africa [34]	*Lancet*
6	644	2020	Prevalence, risk factors, and management of dementia and mild cognitive impairment in adults aged 60 years or older in China: A cross-sectional study [35]	*Lancet Public Health*
7	641	1991	Sleep-disordered breathing in community-dwelling elderly [36]	*Sleep*
8	583	2003	Measuring life-space mobility in community-dwelling older adults [37]	*Journal of the American Geriatrics Society*
9	454	2007	Delay and failure in treatment seeking after first onset of mental disorders in the World Health Organization’s World Mental Health Survey Initiative [38]	*World Psychiatry*
10	437	2005	Assessing mobility in older adults: The UAB Study of Aging Life-Space Assessment [39]	*Physical Therapy*

### 3.6. Keyword Clustering

**Figure 6:**
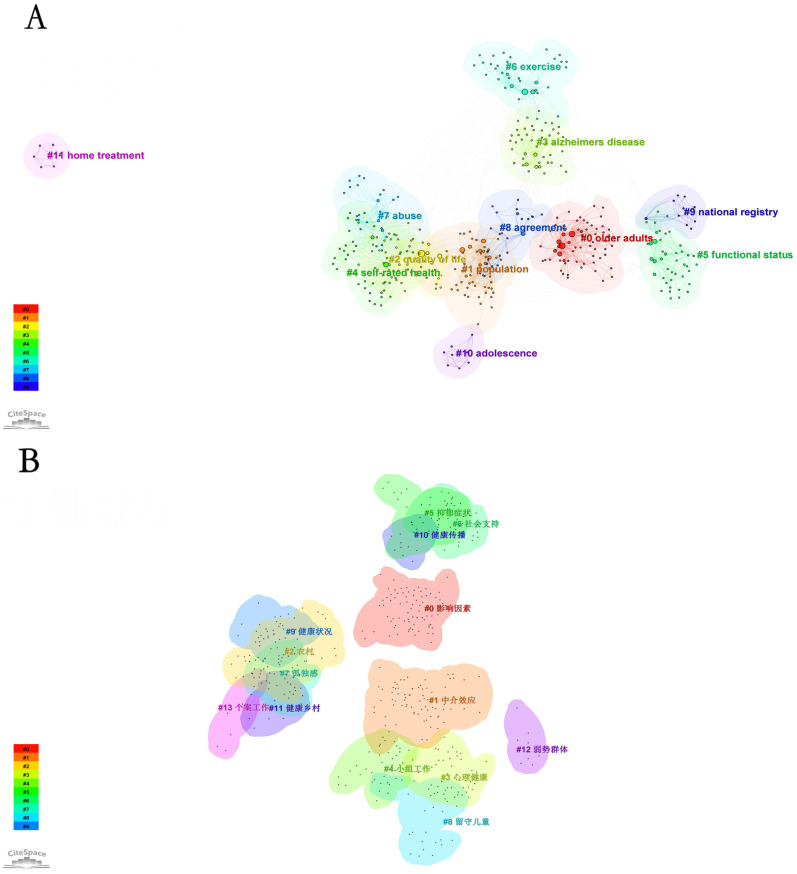
Knowledge map of clustering of keywords in rural elderly mental health research: (**A**) English publications, (**B**) Chinese publications.

Figure 6A illustrates the clustering of keywords in the research field of rural elderly mental health. The main research themes are categorized into several clusters, each representing important research directions in this field. These clusters are marked by keywords such as “Older adults”, “Quality of life”, and “Alzheimer’s disease”, reflecting current research hotspots and trends.

“Older adults” is the largest cluster in the diagram, indicating that mental health research focused on the elderly is a core issue in this field. The focus includes depression, suicidal tendencies, cognitive decline, and other issues closely related to elderly mental health. Social support has a significant impact on the mental health of rural elderly individuals, especially when dealing with life pressures and feelings of loneliness [40]. “Population” examines demographic characteristics and their impact on elderly mental health, exploring factors such as socioeconomic status and living environment. With the rapid urbanization of rural areas in China, many young people migrate to cities for work, leaving a large number of empty-nest elderly individuals facing severe mental health challenges. “Quality of life” emphasizes the positive impact of improving elderly quality of life on mental health, covering intervention measures such as social support and healthcare. Rural elderly individuals with a low socioeconomic status are more prone to mental health issues, closely related to their life pressures and lack of social support [41]. “Alzheimer’s disease” focuses on the impact of this specific disease on elderly mental health, studying early intervention and treatment effects in improving patients’ quality of life.

Other clusters such as “Self-rated health” and “Functional status” explore the subjective assessment of health and the relationship between physical functional status and mental health, indicating that these factors are important predictors and intervention targets for elderly mental health. “Exercise” studies the role of physical exercise in alleviating psychological stress and improving symptoms of depression, while “Abuse” focuses on the issue of elder abuse and its negative impact on mental health. “Agreement” and “National registry” examine the impact of health decision-making consistency on mental health and the application of national data registration systems in large-scale epidemiological studies. “Home treatment” explores the impact of home-based treatment models on elderly mental health, including telemedicine and home care services.

Overall, these clusters highlight the breadth and diversity of research on rural elderly mental health, covering various aspects from medical and psychological treatment to socioeconomic factors, family environment, physical health, and individual subjective assessment. This multidimensional perspective not only reveals the intersections and interactions between different topics but also provides possibilities for integrating research from different fields. Understanding these research clusters can provide scientific evidence for developing more comprehensive and effective mental health intervention strategies and policies, further advancing research and practice in the field of rural elderly mental health.

Figure 6B illustrates the clustering map in Chinese, revealing that research in this field involves multiple interconnected directions, with each cluster representing a different research path. “Influencing Factors” explores how social, economic, and environmental factors impact the mental health of rural elderly individuals. It suggests that rural elderly people with insufficient economic support are more prone to depression and anxiety, with family economic conditions being a crucial factor affecting mental health [42]. “Mediation Effect” delves deeper into the mediating role of these factors and their indirect effects on mental health. “Rural” focuses on the unique challenges posed by rural environments to the mental health of the elderly, such as social isolation and lack of medical resources.

“Mental Health” concentrates on core mental health issues faced by the rural elderly, including depression, anxiety, and loneliness, while “Group Work” examines the effectiveness of group interventions in improving mental health. “Depressive Symptoms” is concerned with the manifestations and impacts of depression, and “Social Support” emphasizes the critical role of social support systems in alleviating psychological stress and promoting mental health.

“Loneliness” investigates the prevalence of loneliness among the rural elderly and its negative impact on mental health, while “Left-behind Children” explores how the situation of left-behind children indirectly affects the mental health of the elderly. “Health Status” and “Health Communication” study the relationship between physical and mental health, and how health communication can enhance health knowledge among the elderly, respectively.

“Healthy Villages” examines strategies for improving the overall health of the elderly through the construction of healthy villages. It notes that the rural elderly have a low level of health literacy, and the lack of health education and mental health services exacerbates their mental health issues [43]. “Vulnerable Groups” focuses on specific psychological health challenges faced by vulnerable groups among the rural elderly. It highlights that rural elderly women are more vulnerable to mental health issues when facing social pressure and economic difficulties, with social support playing a crucial role [44]. Lastly, “Case Work” provides in-depth insights into the mental health problems of the elderly through case studies, offering targeted intervention strategies for individuals.

These clusters comprehensively showcase the breadth and depth of research in this field, covering everything from macro-level social environment analysis to micro-level individual psychological problem interventions. They provide a crucial theoretical foundation and practical guidance for future research and policy-making.

These clusters reflect the broad and in-depth nature of the current research on rural elderly mental health, from environmental and social influencing factors to specific mental health issues and intervention measures. Each cluster displays the interconnections and independence of different research directions within this field. Future research may further integrate these directions to provide more comprehensive and effective solutions to the increasingly complex mental health challenges faced by the rural elderly population.

Table 4 presents the results of keyword clustering analysis for rural elderly mental health research in both the English and Chinese literature, revealing the research hotspots and trends in the field.

In the English literature, the clustering analysis shows that research primarily focuses on elderly populations, demographic characteristics, quality of life, Alzheimer’s disease, and self-rated health. Specifically, “Older adults” is the largest cluster, reflecting the widespread attention given to elderly mental health in recent studies. “Population” highlights the critical role of demographic characteristics in analyzing rural elderly mental health. “Quality of life” demonstrates the importance of improving elderly individuals’ quality of life to enhance their mental health. “Alzheimer’s disease” indicates the profound impact of Alzheimer’s disease on elderly mental health. “Self-rated health” emphasizes the subjective evaluation of health status by elderly individuals as a significant predictor of mental health.

In the Chinese literature, the research focus is more prominently on the unique characteristics of rural settings and the middle-aged and elderly populations. Through cluster analysis, we can observe that “Influencing Factors” is the largest. It primarily explores various influencing factors affecting the elderly population, with the research concentrated around 2013, and a high similarity of keywords within the cluster. In patients with chronic diseases, depression and lack of social support significantly affect their adherence to antiretroviral therapy [45]. “Mediation Effect” shows strong consistency, with research focused around 2016, mainly investigating mediation effects in the context of demographic backgrounds. “Rural” addresses issues related to the quality of life in rural environments, with research concentrated in 2012. “Mental Health” involves the impact of Alzheimer’s disease on mental health. Although this cluster is relatively small, it has lower internal consistency, indicating that the research content may be more diverse. “Group Work” has the highest internal consistency, focusing on strategies to improve elderly individuals’ self-rated health through group work, with research being relatively recent, concentrated around 2017. “Depressive Symptoms” explores the impact of depression on the functional status, with a high degree of similarity between keywords, and the research mainly concentrated around 2013. These clustering results reveal the diversity and complexity of research on rural elderly mental health. The different clusters reflect the concentration of research themes and their temporal distribution, providing important references for understanding the key areas of study in this field and guiding future research directions.

Overall, these clusters reflect different emphases in rural elderly mental health research between the English and Chinese literature. The English literature focuses more on overall health conditions and specific diseases affecting the elderly, while the Chinese literature concentrates on the unique aspects and influencing factors of elderly mental health in rural settings. These differences in research focus provide opportunities for cross-cultural and interdisciplinary studies, highlighting the importance of deepening academic exchange and collaboration globally, particularly in developing intervention strategies and policies to improve rural elderly mental health.

### 3.7. Keyword Clustering Timeline

**Figure 7:**
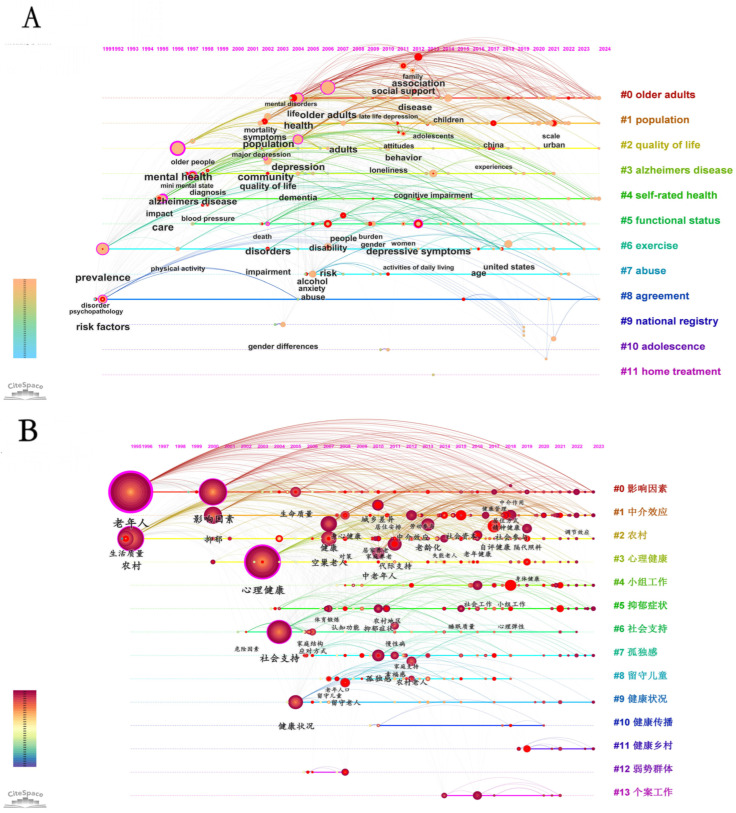
Keyword clustering timeline of rural elderly mental health research: (**A**) English publications, (**B**) Chinese publications.

Figure 7A illustrates the evolution of research themes in the field of rural elderly mental health over time, reflecting the ongoing deepening and development of this area.

From the figure, we can observe that “Older adults” began to gain attention in the early 1990s and has continued to grow as a core research direction over the following decades. The research broadly covers mental health issues among the elderly, such as depression, self-rated health, and quality of life, indicating a sustained focus on elderly health issues. In the context of rapid aging, rural elderly individuals face severe loneliness and mental health challenges due to their children working away from home and a lack of family support [46]. “Population” emerged in the mid-1990s and saw significant growth after 2000, highlighting the important impact of demographic characteristics (such as age, gender, socioeconomic status, etc.) on elderly mental health. The rise of this theme reflects a growing recognition of the importance of demographic factors in mental health research. “Quality of life” started as an independent research theme around 2000 and gradually became a focal point. Researchers have explored how improving social support, medical services, and individual behaviors can enhance elderly individuals’ quality of life and thereby improve their mental health. “Alzheimer’s disease” research increased significantly after 2000, closely related to the global aging problem. This theme focuses on the profound impact of Alzheimer’s disease on elderly mental health, emphasizing the necessity of early diagnosis and intervention.

Additionally, “Self-rated health” and “Functional status” became research priorities after 2000, particularly in examining how elderly individuals’ subjective evaluations of their health and their physical functional status affect their mental health. These studies highlight the critical role of elderly individuals’ perceptions of their health in maintaining mental well-being.

Emerging themes such as “Abuse” and “Agreement” have gained attention in recent years, reflecting the importance of social and legal protections within the elderly population. Meanwhile, “National registry” and “Adolescence”, though less directly related to elderly mental health, highlight the significance of large-scale data registries and cross-generational studies in understanding elderly mental health.

In summary, this timeline clearly illustrates the evolution of research into rural elderly mental health, showing a shift from broad attention to elderly populations towards more specific and specialized themes. Over time, researchers have not only delved deeper into key issues like quality of life, Alzheimer’s disease, self-rated health, and functional status but have also explored diverse aspects such as legal protections, policy implications, and big data technology. This evolving trend provides clear directions for future research and intervention measures, indicating that the field will continue to advance in response to the complexities of global aging.

Figure 7B illustrates the distinct evolution and development trends of themes in the field of mental health research on rural elderly populations across different periods. In the early stages, the focus was primarily on influencing factors and rural environments. These themes emerged early on the timeline and continued to attract research interest, with the increasing size of nodes reflecting the deepening of research in these areas, indicating that they form the foundational research directions in this field. The mental health status of rural elderly individuals is influenced by various factors, including life satisfaction, economic conditions, and social support [47].

As time progressed, the research focus gradually shifted towards themes like mental health and mediation effects. Mental health, as a core theme, has seen an increase in the size of nodes and changes in color, indicating its growing importance in the field. Over the past two decades, in particular, there has been a marked increase in research as societal attention to the mental health issues of the elderly has risen. There are significant differences in the psychological needs of the urban and rural elderly, particularly in social interaction and value needs. Research on mediation effects, although starting later, has quickly become a key research area in the past decade. The development of this node reflects the academic community’s emphasis on understanding the complex mechanisms that influence mental health. A study on the quality of life and influencing factors among elderly Uighurs and Kazakhs in rural Xinjiang aims to provide reference points for improving the quality of life for this population.

In terms of intervention measures, the theme of group work has rapidly emerged in the past decade. The significant increase in the size of its research node on the timeline reflects the growing application and deepening of social work in improving the mental health of rural elderly individuals. Social isolation and lack of social interaction are key factors affecting the mental health of the rural elderly, particularly among those living alone [48]. Meanwhile, research on depressive symptoms and social support has also seen significant growth in recent years. The clear expansion of these themes on the timeline indicates the academic community’s deeper exploration of elderly mental health issues and the role of social support systems. Studies have found that the detection rate of depressive symptoms among rural middle-aged and elderly individuals is closely related to the number of children, the quality of relationships with them, and the frequency of intergenerational interactions.

Notably, the theme of loneliness has seen a significant increase in research nodes following the COVID-19 pandemic, reflecting the profound impact of social isolation on the mental health of the elderly. Loneliness has become an increasingly important research direction. Correspondingly, the theme of health communication has also gained prominence, highlighting its role in enhancing health knowledge and improving mental health among rural elderly individuals. The development of this node indicates the crucial role of information dissemination in health interventions.

Additionally, the themes of healthy rural construction and attention to vulnerable groups have gradually gained more focus over time, with the size of these research nodes increasing, indicating deepening research in these areas. These themes involve broader issues of rural health management and social equity. The distance of healthcare facilities from major medical centers is a significant barrier to access to rehabilitation and other related care for the elderly in rural areas [49]. Case work, a relatively recent research theme, has also begun to attract attention in recent years. The development of its node suggests that the case study approach has unique value in understanding and addressing mental health issues among the rural elderly. In related mental health research, elderly residents in traditional Miao villages are found to be more prone to feelings of loneliness and frustration, which are closely related to their living environment and family structure [50].

Overall, the timeline graph not only displays the starting time and developmental trajectories of various research themes but also reveals the evolution of the research field and shifts in hotspots. Early research primarily focused on the analysis of macro-level influencing factors. However, over time, the focus has gradually shifted towards more specific mental health issues and their intervention measures. This trend reflects the complexity and diversity of mental health research among rural elderly populations, providing a detailed and powerful historical background and trend analysis for future research directions and policy-making. 

### 3.8. Burst Detection of Keywords

**Figure 8:**
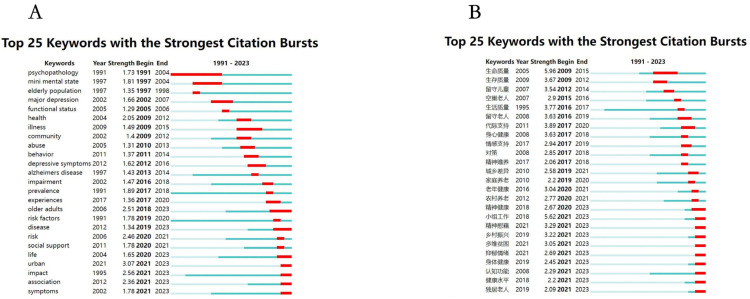
Top 20 most cited keywords in rural elderly mental health research: (**A**) English publications, (**B**) Chinese publications.

Figure 8A presents an English burst detection map showcasing the top 25 keywords in the field of mental health research on rural elderly populations from 1991 to 2023, which experienced the fastest growth in citation frequency, referred to as “burst keywords”. The strength of these keywords and their start and end years demonstrate the significant influence of various research topics during different periods.

Early Burst Keywords (Late 1990s to Early 2000s): Keywords such as “psychopathology”, “mental state”, and “elderly population” began to emerge around 1997 and lasted until approximately 2004. These keywords indicate that in the early stages of research, the academic community focused on the mental health status of rural elderly individuals and the psychological characteristics of the elderly population. “Major depression” and “functional status” emerged in the early 2000s, suggesting that researchers during this period began to pay attention to depression in the elderly and its impact on their functional status.

Mid-Period Burst Keywords (2000s to Mid-2010s): Keywords like “illness”, “community”, and “abuse” emerged significantly between 2004 and 2013, reflecting a shift in research focus from individual mental states to broader social health issues, including the impact of community environments and social support on the mental health of the elderly. “Alzheimer’s disease” and “impairment” emerged between 2007 and 2016, indicating that the research focus during this period gradually expanded to cognitive impairments and their effects on mental health. Access to specialized care varies significantly among elderly patients depending on age, location, and distance from medical centers [51].

Recent Burst Keywords (Late 2010s to Early 2020s): Keywords like “older adults”, “risk factors”, and “social support” began to emerge in the late 2010s and continued until 2023. These keywords reflect a significant increase in attention to quality of life, risk management, and social support for the elderly in recent years. Despite many elderly individuals facing social isolation and mental health issues, they still exhibit high life satisfaction, which may be closely related to community support [52]. Health has a significant impact on labor participation among middle-aged and elderly individuals, particularly in rural areas, where poor health can significantly reduce labor participation rates. After 2020, the emergence of keywords such as “urban”, “impact”, “association”, and “symptoms” highlights the research focus on the impact of urbanization on the mental health of the elderly, and the study of mental health symptoms in the context of new challenges like the pandemic. Widowed rural elderly face higher mental health risks, with the lack of social support being one of the main reasons [53]. The physical and mental health of the elderly should be of great concern, especially for women and those in the western regions of China. Policies to improve the health status of the elderly should be more targeted.

This burst detection map reveals the evolution of mental health research on rural elderly populations. From the late 1990s to the early 2000s, research mainly focused on psychopathology and the mental health status of the elderly population. Over time, the focus gradually expanded to include functional status, community environments, social support, and cognitive impairments. In the late 2010s to early 2020s, the research focus further shifted towards risk management, social support, urbanization, and their impacts on the mental health of the elderly. The socioeconomic differences between urban and rural areas significantly affect the mental health of rural elderly individuals, especially in lower-income families [54]. These burst keywords reflect the dynamic changes in research themes within the field, revealing how social and environmental factors influenced research priorities and hot topics during different periods, providing important directions for future research.

Figure 8B, the Chinese burst detection map, provides a detailed depiction of the citation bursts for the top 25 keywords in the field of mental health research on rural elderly populations, reflecting the evolution and trend changes in research hotspots from 1991 to 2023. By analyzing these keywords, we can observe how the focus of research gradually shifted from broad health and quality of life issues to more specific mental health topics and the needs of special groups.

Early Burst Keywords (Late 2000s to Early 2010s): Keywords such as “quality of life” (starting in 2005, with a strength of 5.96, lasting until 2015) and “survival quality” (starting in 2009, with a strength of 3.67, lasting until 2012) were prominent during this period. In rural areas, the social engagement of the elderly is significantly positively correlated with their mental health status, with the lack of community support exacerbating this issue [55]. The research focused on the quality of life and survival status of the elderly, indicating the academic community’s concern for improving the overall living standards of the elderly. Keywords such as “left-behind children” (starting in 2007, with a strength of 3.54, lasting until 2014) and “empty-nest elderly” (starting in 2007, with a strength of 2.9, lasting until 2016) reflected the mental health issues of the rural elderly due to changes in family structure, such as children going out to work. Research on the sleep quality of the rural elderly revealed that adverse childhood experiences are an important influencing factor, affecting sleep quality indirectly through anxiety and negative coping mechanisms [56]. In rural China, the mental health status of the widowed elderly is significantly poorer, with higher rates of depression and loneliness compared to those with spouses [57].

Mid-Period Burst Keywords (Mid-2010s to Late 2010s): Keywords like “left-behind elderly” (starting in 2016, with a strength of 3.63, lasting until 2019) and “intergenerational support” (starting in 2011, with a strength of 3.89, lasting until 2020) reflected the academic community’s focus on changes in social support networks for the rural elderly, especially how elderly individuals maintain mental health through intergenerational support in the context of children working away from home. The high prevalence of loneliness among the rural elderly is closely related to their lack of a social support system [58]. “Emotional support” (starting in 2017, with a strength of 2.94, lasting until 2019) and “spiritual support” (starting in 2017, with a strength of 2.06, lasting until 2018) emphasized the importance of emotional connections and psychological comfort among the elderly population. Social isolation and lack of social interaction are significant factors affecting the mental health of the rural elderly, particularly among those living alone [59].

Recent Burst Keywords (Late 2010s to Early 2020s): In recent years, keywords like “elderly health” (starting in 2016, with a strength of 3.04, lasting until 2021) and “mental health” (starting in 2018, with a strength of 2.67, lasting until 2023) reflect the continued focus on the overall health and mental health of the elderly. There is a positive link between elderly participation in religious activities and their mental health, particularly in rural areas [60]. Especially in the post-pandemic era, mental health issues have become a prominent research topic. Managing hypertension among the rural elderly poses significant challenges, impacting both their physical and mental health [61]. “Group work” (starting in 2018, with a strength of 5.62, lasting until 2023) shows the increasing application of social work interventions in improving mental health among the rural elderly and has become a key research direction in recent years. It has been shown that the Mediterranean diet has protective effects against age-related diseases, as validated in the longevity of the populations of Sicily [62].

Latest Burst Keywords (Early 2020s to Present): Keywords such as “spiritual comfort” (starting in 2021, with a strength of 3.29, lasting until 2023) and “rural revitalization” (starting in 2021, with a strength of 3.22, lasting until 2023) indicate researchers’ recent focus on diversified psychological interventions and mental health in the context of rural revitalization. Particularly in the absence of social support, symptoms of depression are more pronounced. The inadequate health services in rural areas result in many elderly individuals not receiving timely psychological health interventions, exacerbating their mental health issues [63]. “Depressive emotions” (starting in 2021, with a strength of 2.69, lasting until 2023) and “cognitive function” (starting in 2021, with a strength of 2.29, lasting until 2023) reflect the latest academic attention to cognitive decline and depression among the elderly, particularly among those living alone. The quality of life of the rural elderly is closely related to their mental health status, with a significantly higher incidence of depression among those with lower social participation [64].

This burst detection map reveals the evolution of the research focus and hotspots in the field of mental health for rural elderly populations. Early research was centered on the quality of life and survival quality of the elderly, as well as mental health issues arising from changes in the family structure. Over time, research gradually delved into more specific areas of mental health support, including social support, emotional support, and spiritual comfort. In recent years, the focus has further shifted to comprehensive management of elderly mental health, including cognitive decline, depression management, and the application of social work. These burst keywords show how research directions have adjusted with changes in social environments and rural revitalization policies, providing valuable insights for future research and intervention measures.

## 4. Discussion

This study provides a comprehensive bibliometric analysis of research on the mental health of rural elderly populations, uncovering significant trends and evolving research priorities over the past decades. By employing CiteSpace, we identified the growing attention to mental health issues within this vulnerable group, reflecting their increasing importance in both domestic and international academic contexts. The findings reveal distinct patterns in research focus, collaboration networks, and emerging themes, offering valuable insights into the trajectory of this field.

The results highlight the strong research activity in China, driven by the challenges of an aging population and the unique socioeconomic conditions of rural areas. The focus of domestic studies on specific issues, such as the mental health of empty-nest elderly individuals [65,66], the role of intergenerational support [67], and the effects of rural living conditions [68],underscores the need for targeted interventions. Internationally, research themes tend to prioritize broader health outcomes, such as quality of life [69] and the impact of chronic diseases [70], suggesting differing perspectives and priorities between domestic and global studies.

An important finding of this study is the evolution of research themes. Early studies predominantly explored foundational psychological issues, such as loneliness and depression [71,72], while more recent research has expanded to include the impacts of urban-–ural integration [73], access to mental health services, and policy interventions [74,75,76,77]. This shift indicates an increasing recognition of the complex interplay between social, economic, and health-related factors influencing the mental health of rural elderly populations. However, the limited collaboration between Chinese and international researchers reflects a missed opportunity for knowledge exchange and innovation, as emphasized in previous studies addressing global aging challenges.

The study also identifies research hotspots, such as the role of social and family support in improving mental health outcomes [78,79]. These findings align with the growing consensus in the literature that strong social networks and community engagement are crucial for mitigating mental health risks in the elderly. Additionally, the identification of emerging themes, such as the influence of digital health technologies [80,81] and rural revitalization policies [82], provides a forward-looking perspective on future research directions.

Overall, this study contributes to the field by systematically summarizing the state of research on rural elderly mental health, highlighting key achievements while identifying areas requiring further exploration. The findings serve as a foundation for advancing the academic discourse and inform the development of practical interventions and policies tailored to the needs of rural elderly populations.

## 5. Conclusions

### 5.1. Strengths and Advantages

One of the primary strengths of this study is the application of CiteSpace, which allowed for a dynamic and in-depth analysis of bibliometric data. Unlike traditional methods, CiteSpace’s advanced visualization capabilities, including citation burst detection and thematic clustering, provided a nuanced understanding of the research field’s evolution. These capabilities provide a more comprehensive understanding of research evolution compared to tools like VOSviewer or HistCite. This methodology enabled the identification of emerging research themes and collaboration networks, offering insights that are both comprehensive and actionable.

Additionally, this study bridges domestic and international research perspectives, providing a balanced view of the field. By integrating findings from WOS and CNKI, the analysis captures both global trends and localized insights, enhancing this study’s relevance and applicability. This dual perspective is particularly valuable for addressing the challenges faced by rural elderly populations, whose mental health issues are shaped by unique cultural and socioeconomic contexts.

### 5.2. Limitations and Future Directions

Despite its contributions, this study has certain limitations. First, the reliance on the WOS and CNKI databases may have excluded relevant studies from other sources, such as Scopus or regional repositories. This limitation may impact the generalizability of the findings, particularly in non-Chinese contexts. Second, the bibliometric approach, while robust in identifying trends and patterns, lacks the qualitative depth needed to explore the underlying mechanisms driving these patterns. Future research should integrate qualitative methods to capture the lived experiences of rural elderly individuals. Lastly, terminological variations between Chinese and English research contexts may have introduced biases in data retrieval and interpretation.

Future studies should address these limitations by incorporating additional databases, expanding cross-cultural collaborations, and employing mixed-methods approaches. Interdisciplinary research, integrating perspectives from sociology, public health, and digital technologies, is particularly needed to address the multifaceted nature of rural elderly mental health.

### 5.3. Practical and Policy Implications

The findings of this study have significant implications for social and healthcare policy. Policymakers should prioritize interventions that strengthen intergenerational and community support systems, as these are critical for improving mental health outcomes [83]. For example, programs that promote financial and emotional connections between urban migrant workers and their elderly parents in rural areas can mitigate the adverse effects of loneliness and depression.

Additionally, this study underscores the need for tailored mental health services in rural areas. Investments in community-based care, telemedicine, and digital health technologies can bridge the gap in service accessibility, particularly for isolated elderly populations. The role of rural revitalization policies in creating supportive environments for elderly mental health should also be explored further.

Finally, international collaboration must be strengthened to address the global nature of aging-related mental health issues. Partnerships between Chinese and international researchers can foster innovation, knowledge exchange, and the development of culturally adaptable interventions. These efforts are essential for advancing the field and ensuring the well-being of rural elderly populations worldwide.

## Figures and Tables

**Table 1:** Top 5 institutions that contributed to the publications.

English Publications					Chinese Publications				
Rank	Count	Centrality	Year	Institution	Rank	Count	Centrality	Year	Institution
1	79	0.06	1999	University of London	1	65	0	2014	山东大学 Shandong University
2	42	0.15	2004	US Department of Veterans Affairs	2	39	0	2019	西南财经大学 Southwestern University of Finance and Economics
3	41	0.06	2005	King’s College London	3	24	0	2017	中南财经政法大学 Zhongnan University of Economics and Law
4	29	0.13	2004	Veterans Health Administration (VHA)	4	24	0	2014	吉林大学 Jilin University
5	25	0.22	2005	Peking University	5	22	0	2015	华中科技大学 Huazhong University of Science and Technology

**Table 2:** Top 5 countries that contributed to the publications.

English Publications				
Rank	Count	Centrality	Year	Country
1	342	0.13	2000	PEOPLE’S R CHINA
2	226	0.64	1991	USA
3	172	0.78	1997	ENGLAND
4	96	0.19	1999	CANADA
5	82	0.25	2003	AUSTRALIA

**Table 4:** Clustering labels of keywords in rural elderly mental health research publications (English and Chinese).

ClusterID	Size	Silhouette	Mean (Year)	Label (LLR)	ClusterID	Size	Silhouette	Mean (Year)	Label (LLR)
0	68	0.691	2015	older adults (11.67, 0.001)	0	80	0.684	2013	影响因素 (152.2, 1.0 × 10^−4^) influencing factors
1	61	0.697	2015	population (7, 0.01)	1	77	0.756	2016	中介效应 (51.64, 10 × 10^−4^) mediation effect
2	50	0.782	2010	quality of life (8.32, 0.005)	2	62	0.683	2012	农村 (94.75, 1.0 × 10^−4^) rural
3	45	0.828	2006	Alzheimer’s disease (11.39, 0.001)	3	47	0.576	2012	心理健康 (171.9, 1.0 × 10^−4^) mental health
4	43	0.778	2011	self-rated health (11.17, 0.001)	4	35	0.858	2017	小组工作 (62, 1.0 × 10^−4^) group work
5	43	0.825	2011	functional status (9.3, 0.005)	5	34	0.774	2013	抑郁症状 (24.46, 1.0 × 10^−4^) depressive symptoms

## Data Availability

Data will be available from the corresponding author (Lei Yang) on request.
